# The Influence of Body Position on Determining Aerobic Exercise Intensity for Healthy Individuals

**DOI:** 10.3390/ijerph182211773

**Published:** 2021-11-10

**Authors:** Rogério de Siqueira Peters, Maria do Socorro Luna Cruz, Claudio Hernández-Mosqueira, Cristian Martinez-Salazar, Fernando Policarpo Barbosa

**Affiliations:** 1 Secretariat of Education of the Federal District, Brasilia 72210-140, Brazil; rog.peters30@gmail.com; 2 Faculdade de Ciências Médicas-FCM/CG, Universidade Federal do Rio Grande do Norte (UFRN) FACISA Campus, Santa Cruz 59200-000, Brazil; socorrolcruz@gmail.com; 3 Departamento de Educación Física, Deportes y Recreación, Universidad de La Frontera, Temuco 4780000, Chile; cristian.martinez.s@ufrontera.cl; 4 Laboratory of Bioscience of Human Movement, Federal University of Rio de Janeiro, Rio de Janeiro 21941-901, Brazil; fernandopolicarpo65@gmail.com

**Keywords:** prescription, aerobic training, heart rate, young people, healthy

## Abstract

To verify the influence of the resting heart rate (RHR) measurement on different positions in the calculation of VO_2max_ intensities in young individuals of both sexes. Methods: A cross-sectional study with a correlational design with a sample of 22 men and 11 women, aged 27.8 ± 6.5 years and 29.0 ± 8.6 years, respectively, healthy, active and sedentary, who performed the stress test on a treadmill until fatigue. For the treatment of the data, a repeated measures ANOVA was carried out with Bonferroni’s post hoc test. Results: The comparison of the mean values of baseline heart rate (Bhr) between the groups of women and men showed significant differences (t = 2.329; gl 31; *p* = 0.027). However, no significant differences were noted for lying (t = 0.057; gl 31; *p* = 0.95), sitting (t = 0.196 gl 31; *p* = 0.85) or standing (t = −0.290; gl 31; *p* = 0.77). But in the analysis of the intensities of the RHR in different positions, the calculations with baseline and lying HR were significantly different (*p* < 0.05). Conclusion: The determination of aerobic training intensities by RHR method must observe the heart rate measurement at rest in the sitting and/or standing positions minutes before the training session.

## 1. Introduction

The determination of the intensities of the percentage of maximal oxygen consumption (VO_2max_), oxygen consumption reserve (VO_2_R) and heart rate reserve (HRR) for aerobic training has been called into question, i.e., the relation of linearity between heart rate (HR) and oxygen consumption during effort tends to under- or overestimate the intensity of aerobic exercise [1,2]. However, there are other physiological factors related to heart rate monitoring which may clarify some of the issues [3,4,5,6].

One such factor is the position of the body at the time of the resting heart rate (RHR) measurement, such as in the lying position, in which venous return is favored. Fisher et al. [7] explain that the HR is lower when performing dynamic exercises in the supine position compared to the upright position, which indicates the existence of mechanisms that induce changes in central blood volume and cardiopulmonary load that can influence the cardiovascular response during static or dynamic exercise in humans. Another factor that may interfere with the results presented in the studies is the decision to use the value of 3.5 mL/kg·min^−1^ of oxygen as the standard value for oxygen consumption at rest in the calculation of VO_2_R [8]. According to Méndez-Villanueva et al. [9], the body mass involved in physical effort can also present significant differences between the % of VO_2max_ and the % of VO_2_R when they are associated with the % of HRR.

Both the hemodynamic and hormonal factors (catecholamine concentrations) and the response of or adjustments by the autonomic and peripheral nervous system are important to HR adjustment during exercise [7,10,11]. Birnbaumer et al. [3] describe sex and age as being directly related to the reduction in maximum HR and maximal power output, which is consistent with previous studies related to the loss of physical fitness with age [5,12]. This may explain the critical linear relation between % VO_2max_ and % HRR or % VO_2_R and % HRR, since there is no way to keep the oxygen consumption stable during continuous exercise [3]. 

In this sense, the aim of this study is to verify the influence of measuring the resting heart rate (HRr) in different positions in the calculation of intensities: 50, 60, 70 and 80% of VO_2max_ through the reserve heart rate equation (HRR) in young men and women as recommended by the American College of Sports Medicine.

## 2. Materials and Methods

This is a cross-sectional study with a correlational design [13]. The sample was made up of 22 men and 11 women, mean age 27.8 of ± 6.5 years and 29.0 ± 8.6 years, respectively, healthy, active and sedentary. The individuals were interviewed by the researcher to obtain the level of weekly practice of physical exercises and/or physical activities and; according to the guidelines of the American College of Sports Medicine [14], those who reported regular levels of physical exercise lasting at least thirty minutes a day were classified according to the hours dedicated to exercise and/or physical activities per week, three to five times a week for six months or more of training. and sedentary, those who did not perform less than 150 min of weekly physical activities [14]. These subjects participated in the study through an invitation made to employees of a university and a gymnasium in the Federal District of Brazil. The present study complied with the recommendations for studies with human beings described in resolution 446/12, being presented and approved by the protocol of the Ethics Committee of the Catholic University of Brasilia (UCB) on 20 June 2005, number 045/2006.

After the invitation, everyone received information on the procedures, as well as the possible discomforts, risks and benefits of the cardiopulmonary stress test. Those who agreed to participate were told their baseline heart rate (Bhr) would be measured within five days; the measurement took place on waking. The individual could not make movements such as sitting or getting up from the bed before measuring the heart beats using the method of pressing on the radial artery. The count was taken in one minute, considering the average of five days prior to the day of the cardiopulmonary exercise test. The day before the cardiopulmonary stress test, the following procedures were implemented: (1) no vigorous physical exercise twelve hours before the cardiopulmonary exercise test; (2) no drinking alcohol or caffein; and (3) eating light food at least two hours before exercise test [15,16].

When the volunteers came in to the laboratory on the scheduled date and time, they were again informed of the procedures and they signed a free and informed consent. Next, they responded to the Par-Q, and body mass and height measurements were taken using the Filizola digital scale (Personal Line model) accurate to 100 g for weight, and the stadiometer of the same scale was used for height in centimeters. Then, the individuals stayed lying down for five minutes before their blood pressure was taken by auscultation, using the Becton Dickinson^®^ sphygmomanometer (Becton Dickinson, São Paulo-SP, Brazil). After this, an electrocardiogram was taken at rest with the Marquette Hellige, Medical Systems, model: CardioSmart© v. 3.0 CS-MI (General Electric Company, Boston, MA, USA).

Once medical authorization had been obtained, the RHR evaluation began. The volunteers were transferred to a temperature-controlled room with smooth environmental light, where they were monitored by the Micromed^®^ electrocardiogram (MicroMed, Brasilia, Brazil) using three CM5 leads, and they stayed lying down on a gurney. Heart rate (HR) was measured for five minutes in each position: lying (HR_lying_), sitting (HR_sitting_) and finally standing (HR_standing_). After the change of position, it was expected that the HR would stabilize before beginning a new reading. Then, the cardiopulmonary exercise test was performed on a treadmill with gas analysis using the VO_2_000^®^ by Aerosport Medgraphics (Oak Grove, MN, USA) to determine the peak oxygen consumption (VO_2_peak), with the collection of expired gases monitored every 10 s. The metabolic gas analyzer was calibrated before each test with a known gas concentration (17% oxygen-O2 and 5% carbon dioxide-CO_2_), following the manufacturer’s recommendations.

To determine the VO_2_peak and HRmax, the volunteers performed an incremental test on an Inbramede^®^ Super ATL (Inbramede, Porto Alegre, Brazil) treadmill until fatigue. The staggered protocol had an initial speed of six km/h with zero percent of incline and a final speed of 16 km/h with 6% of incline; being observed the interval of one minute for the increments [15]. The electrocardiogram, the treadmill, and the gas analyzer were controlled by the Micromed^®^ ErgoPC Elite software v. 3.2.1.11 (MicroMed, Brasilia, Brazil). After the test, first and second ventilatory thresholds were marked according the recommendations of Policarpo et al. [16] and Grigaliuniene et al. [17].

After the test, the second ventilatory threshold was determined, considering it a point to determine an abrupt increase in expiratory volume greater than 8 L/min. The following parameters were adopted as tie-breaks: (a) an exponential increase in ventilatory equivalent for oxygen-VE/VO_2_, with stabilization or increase in the ventilatory equivalent for carbon dioxide-VE/VCO_2_ [18] and (b) a drop in the expired carbon dioxide fraction-FeCO_2_ [1].

At the end of the stress test, the intensities of 50, 60, 70 and 80% of the VO_2_peak and HR were calculated corresponding to the respective intensities. This calculation was determined by the per-minute mean. Then, the intensities were calculated using the equation tHR = RHR + [(HRmax − RHR) ×%] [19], with the training heart rate (tHR) and the variation in the calculation of the baseline heart rate (Bhr), lying (HR_lying_), sitting (HR_sitting_) and standing (HR_standing_) heart rates.

### Statistical Analyses

For a more detailed analysis of the two pieces of data, a dispersion curve was created from the data on the 33 volunteers, and then the dispersion curve concerning the physical condition of the active and sedentary volunteers was analyzed using the Kolmogorov-Smirnov test and the QQ plot of normality. The results indicated a normal distribution curve at *p* < 0.05. Student’s t test for independent samples was applied to analyze anthropometric and physiological variables in physically active and sedentary subgroups. The mean HR corresponding to the intensities of 50, 60, 70 and 80% of the VO_2_peak were compared with the values calculated by the tHR equation for all conditions. Therefore, the repeated measures ANOVA was applied, respecting the assumption of sphericity of Mauchly’s test. When statistically significant differences between the measures were observed, Bonferroni’s post hoc test was applied. The significance level in all the analyses was set at *p* < 0.05.

## 3. Results

The sample was made up of 33 volunteers, 11 were women, 4 sedentary and 7 physically active, with a mean age of 27.00 ± 3.67 years and 31.14 ± 9.23 years, respectively. The men’s group had 8 sedentary men, with an average age of 26.63 ± 5.78 years and 14 physically active ones, with a mean age of 28.36 ± 7.03 years. The comparison of the mean values by age showed no statistical difference (t = −0.849 for fd = 9; *p* = 0.42) between sedentary and physically active women. A similar behavior was noted for the age of the sedentary and physically active men (t = −0.596 for fd = 20; *p* = 0.56) Table 1.

The analysis of the anthropometric parameters points to the homogeneity in women’s and the men’s group, since no statistical differences were observed for body mass (t = 0.959 for fd = 9; *p* = 0.42), height (t = 0.430 for fd = 9; *p* = 0.68) or for the BMI (t = 0.962 for fd = 9; *p* = 0.36) between the sedentary and physically active women. There was no difference in body mass between the sedentary and physically active men either (t = −1.234 for fd = 20; *p* = 0.23), height or BMI (t = −1.318 for fd = 20; *p* = 0.20) and (t = −0.950 for fd = 20; *p* = 0.56).

**Figure 1:**
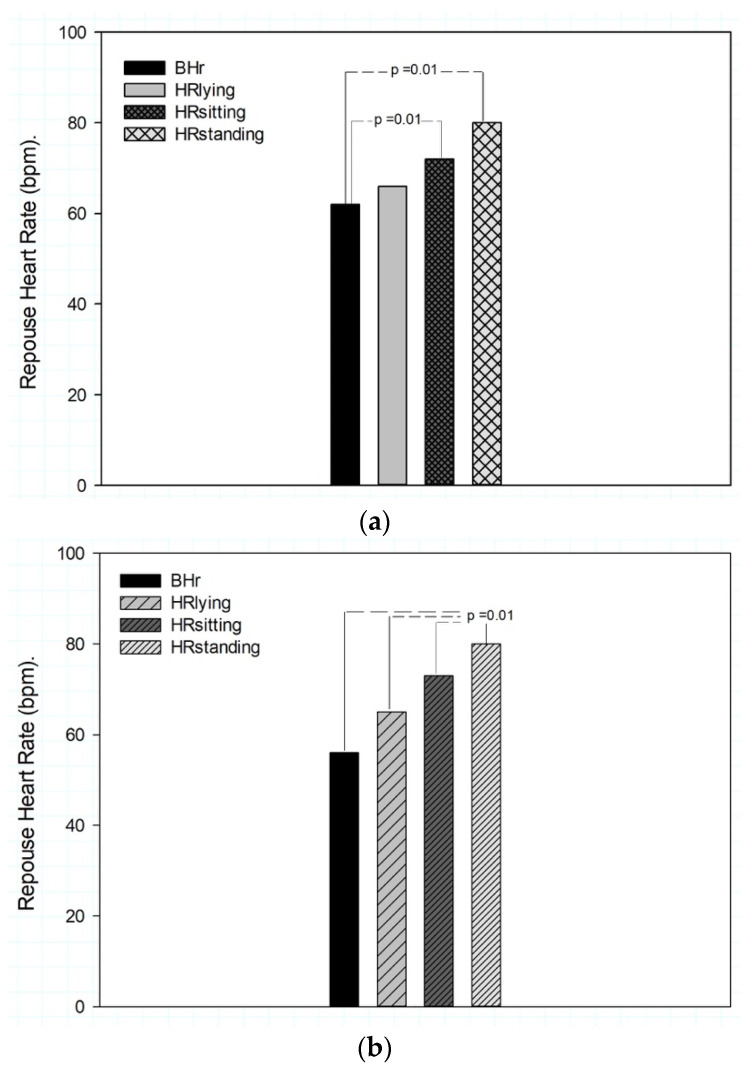
Baseline and resting heart rate measured in different positions. (**a**) average values of baseline and resting heart rate in different positions in 11 young women; (**b**) average values of baseline and resting heart rate in different positions in 22 young men.

The comparison of the mean Bhr values between the groups of women and men yielded significant differences (t = 2.329; fd = 31; *p* = 0.027) between the groups. However, there were no significant differences for lying (t = 0.057; fd = 31; *p* = 0.95), sitting (t = 0.196; fd = 31; *p* = 0.85) or standing (t = −0.290; fd = 31; *p* = 0.77) Figure 1. As illustrated in Table 2, the statistical results for the resting states indicated no significant differences for the HR between the groups of women and men in the lying, sitting or standing positions. A similar behavior was obtained based on the level of physical condition, in which there was no significant difference between the sedentary and physically active groups when the baseline and resting heart rate were compared.

However, the results of the comparison of the sedentary and physically active subgroups did not show any statistical difference. This is because the RHR analysis in the women’s group showed a significant difference in Wilk’s Lambda F (3,17.684) = 8.000; *p* = 0.01 for an alpha of 0.99 between Bhr and HR_lying_ compared to HR_sitting_ and HR_standing_. Significant differences were also observed in Wilk’s Lambda F (3, 31.935) = 0.165; *p* = 0.001 for all the positions in the men’s group, which indicates a greater variation in HR for this group in all the analyzed positions (Figure 1).

The coefficient of variation (CV) obtained for the basal heart rate of women was 8.79%; in the other positions: lying down = 17.13%; sitting = 15.05% and standing = 11.13%. CV values for men in the respective positions were: = 13.11% and 13.54%; 16.35% and 15.80%. The values obtained for the CV indicate a trend of homogeneity, since the fluctuation of the data is less than 5% for both women and men. However, CV values in the men’s group may explain the statistical difference observed in the resting state.

No difference was noted in oxygen consumption: 21.56 ± 5.96 mL/kg·min^−1^ vs. 24.56 ± 6.06 mL/kg·min^−1^ in the first ventilatory threshold between the groups of women and men, or for heart rate: 133 ± 13 and 124 ± 16 bpm, respectively. In addition, there were no differences for the HRmax in the maximum effort between the groups: women = 183 ± 9 bpm and men = 187 ± 9 bpm. However, the mean oxygen consumption values in the second ventilatory threshold for the respective groups: women = 36.49 ± 7.12 mL/kg·min^−1^ and men = 43.65 ± 5.78 mL/kg·min^−1^ were significantly different (t = −3.103; fd 31; *p* = 0.01), as well as for VO_2_ (t = −4.444; fd 31; *p* = 0.01). At the peak of exercise, they presented a statistically significant difference when the groups of women and men were compared: 40.62 ± 5.21 mL/kg·min^−1^ vs. 51.86 ± 7.51 mL/kg·mni^−1^, respectively.

The values for heart rate and oxygen consumption in relation to the second ventilatory threshold of the women were ≈53% of the RHR, calculated using the formula: HR = (training HR − resting HR)/(HRmax − resting HR). Since the CV was 9.75% for heart rate and 27.64% for oxygen consumption, there is a broader spread of data in this variable. The second ventilatory threshold is 90.74% of HRR and corresponds to 89.83% of the oxygen consumption, with the CV being 4.47% and 19.51% respectively. In the men’s group, the first ventilatory threshold was 42.34% of HRR with a CV = 13.26%, whereas the oxygen consumption was 47.35% with a CV = 24.67%. The second ventilatory threshold values showed a narrower spread, being 87.39% of the HRR and 84.69% of the peak VO_2_, with a CV = 5.62% and 13.24% respectively.

**Figure 2:**
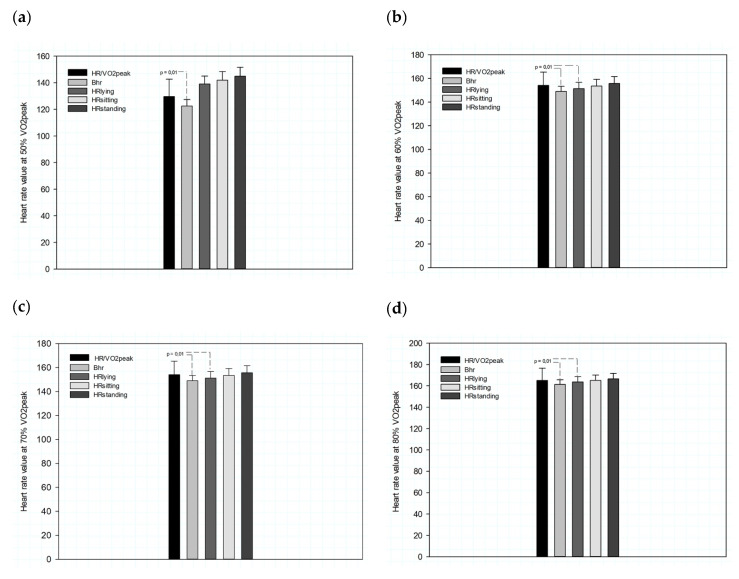
Baseline and resting heart rate measured in different positions for intensities 50, 60, 70 and 80% of the peak oxygen consumption and the respective heart rate (HR/VO_2_peak). Charts (**a**–**d**) show the values for intensities 50, 60, 70 and 80% of peak oxygen consumption and the respective heart rate (HR/VO_2_peak) and for the intensities calculated by resting heart rate in the positions: baseline (Bhr), lying (HR_lying_), sitting (HR_sitting_), and standing (HR_standing_) for a sample of young individuals of both sexes.

For a deeper analysis, it was decided to group the data and verify if there were any differences, since the equation of heart rate reserve does not discriminate between sexes. Thus, the data show a statistical difference F (4, 26.371) = 29.000; *p* = 0.01 for 50% and 60% intensity when it is calculated using the Bhr position. At intensities of 70% and 80%, a difference was also found for the values calculated in the HR_lying_ position. The differences encountered refer to the comparison with the reference values, i.e., the mean HR relative to the oxygen consumption by intensity. There is also a significant difference F (4, 26.730) = 29.000; *p* = 0.01 between the values calculated with Bhr and HR_lying_ when they are compared with the values calculated for HR_sitting_ and HR_standing_ (Figure 2).

## 4. Discussion

The objective of this study was to verify the influence of the FCR obtained in different positions in the calculation of intensities through the FCR and, at the same time, to verify which would be more accurate in determining the percentages of VO_2max_. Figure 2 shows the mean values of the intensities of 50, 60, 70 and 80%, based on Bhr, HRlying, HRsitting and HRstanding as the basis for the calculations.

Among the limiting factors of the study, the lack of experience/ability of the volunteers to measure the basal heart rate by the radial artery palpation method stands out, as well as the consistency in following a protocol for measuring heart beats on awakening, these two factors may have compromised the quality of the data to the variable in question. Another limiting factor would be the small number of sedentary individuals in the sample. However, the smaller number of sedentary individuals is not an intervening factor that can compromise the observed results, since there is no difference in the calculation of aerobic training intensities for individuals with different levels of physical fitness [4,5].

The relevant point of the present study is in the direct application of the results at the time of prescription of aerobic training intensities, since there is no difference in the measurement of resting heart rate in the sitting or paddle positions. At the same time, the results allow us to question previous studies that did not take into account the adjustments induced by the nervous system both in oxygen consumption and in heart rate [1,3,12].

The results obtained in the present study clearly describe that body position influences the determination of intensities corresponding to the percentages of VO_2max_. This is directly due to the reduced homedynamic load favoring venous return as described by Fisher et al. [7]. This is a phenomenon also observed during aquatic activities, wave at the depth at which the exercise is performed tends to reduce heart rate [20].

The variation in heart rate values in the different positions as mentioned above represents the hemodynamic adjustments, which increase the systolic volume and, consequently, reduce the heart rate [4,7,12,21]. At the same time, it is possible to observe a reduction in sympathetic action and a reduction in peripheral resistance due to the low concentrations of circulating catecholamines, which facilitate venous return [4,7]. This phenomenon is evident among physically active individuals [21], as the cholinergic responses in these individuals undergo adaptations due to training [4]. As can be seen in Figure 2, the use of basal HR and HR at rest (lying down) resulted in lower values in the determination of intensities, especially in the intervals of 50 and 60% of peak VO_2_, tending to underestimate the training loads, as reported in previous studies [2,16]. In practical terms, it is important that when prescribing aerobic training using the oxygen consumption reserve equation (VO_2_R), the professional observes the measurement of heart rate in one of the sitting or standing positions, this may mitigate the VO_2_R error in overestimation, as well as, of the HRR in underestimating the intensities Policarpo et al. [1].

However, it should be emphasized that intensities below 60% of VO_2_peak are more susceptible to the parasympathetic autonomic system [3,4,21], which explains the heart rate variability observed at intensities of 50 and 60% of VO_2_peak [7] pointed out in the results presented here. The variability observed is described in the notes by Åstrand et al. [22], where the loss of linearity between oxygen consumption and heart rate is emphasized. This loss of linearity may be related to the action of physiological mechanisms (chemoreceptors, baroreceptors and mechanoreceptors) that make adjustments in HR in relation to metabolic residues and muscle tension [4,7,20,23,24]. 

In the study by Policarpo et al. [1] and Iannetta et al. [25] they observed disparities between the percentages of VO_2max_ and those determined by indirect methods (HRR and VO_2_R). This is consistent with the conclusions of Ferri Marini et al. [26] and earlier such as [8]. What could explain this loss of linearity would be the adjustments described by Åstrand et al. [22] due to metabolic changes induced by exercise intensity. The present study did not aim to analyze the association between the percentages of HRR and VO_2max_. However, the values obtained for the studied percentages did not show statistically significant differences as shown in Figure 2.

The results obtained in this study point to the need for complementary studies aimed at determining the intensities, since the methods applied for the prescription of aerobic training intensities have some limitations [3,25]. Policarpo et al. [1] point out the need for specific equations for each training intensity. Consistent with the notes by Ferri Marini et al. [26] which cites, that both the HRR and the VO_2_R differ from the line of identity; the authors add that the use of a single equation may not be appropriate to determine aerobic exercise intensity. This suggests the need to rethink the relationships between the methods for determining the intensity of aerobic exercise, in order to ensure greater efficiency and safety in training prescription.

## 5. Conclusions

The results of the present study allow us to conclude that the determination of aerobic training intensities by the FCR method will have the expected acuity when the resting heart rate measurement is performed in the sitting and/or standing positions. For this, professionals must do it 5 min before the training session. At the same time, the need for future studies to develop specific indirect methods for each aerobic training intensity is evident, since there is evidence of loss of linearity between heart rate and oxygen consumption during aerobic exercise.

## Figures and Tables

**Table 1:** Results of the anthropometric characteristics of young men and women who underwent the maximum stress test on a treadmill.

		Body Mass (kg)	Height	BMI (kg/m^2^)
		Mean ± SD	Mean ± SD	Mean ± SD
Women	Sedentary (n = 4)	63.72 ± 7.45	161.5 ± 11.59	24.60 ± 3.59
	Active (n = 7)	57.78 ± 10.89	159.5 ± 3.90	22.60 ± 3.17
Men	Sedentary (n = 8)	73.36 ± 11.24	173.19 ± 5.30	24.40 ± 3.00
	Active (n = 14)	80.12 ± 12.92	176.06 ± 4.68	25.76 ± 3.36

SD: Standard deviation.

**Table 2:** Description of the heart rate measurement in different positions in young individuals of both sexes.

	Women		Men	
Positions	Sedentary	Active	Sedentary	Active
	Mean ± SD	Mean ± SD	Mean ± SD	Mean ± SD
Bhr	59.5 ± 4.5 *	61.3 ± 6.0 ‡	55.1 ± 5.2	54.8 ± 8.3
HR_lying_	68.7 ± 13.4	61.1 ± 9.2	61.4 ± 8.0	61.9 ± 8.8
HR_sitting_	77.2 ± 11.9	67.1 ± 8.7	73.2 ± 12.3	68.6 ± 11.1
HR_standing_	75.0 ± 9.1	74.4 ± 8.9	76.7 ± 10.3	76.6 ± 13.4

HR: heart rate; Bhr: baseline heart rate; HR_lying_: lying; HR_sitting_: sitting; HR_standing_: standing; SD: standard deviation; * = *p* > 0.05 Compared to the sedentary male group; ‡ = *p* > 0.05 Compared to the physically active male group.

## Data Availability

The underlying research materials related to this paper are available from the corresponding author upon request.

## References

[1] Policarpo F., Valdivia-Moral P., Luna-Cruz M.D.S., Véliz-Burgos A., Mansilla-Sepulveda J., Estay-Sepulveda J.G. (2019). Comparison between Different Prescription Methods for Aerobic Training in Young Adults. Sustainability.

[2] Rolnick N., Schoenfeld B.J. (2020). Can Blood Flow Restriction Used During Aerobic Training Enhance Body Composition in Physique Athletes?. Strength Cond. J..

[3] Birnbaumer P., Traninger H., Borenich A., Falgenhauer M., Modre-Osprian R., Harpf H., Hofmann P. (2020). Heart Rate Performance Curve Is Dependent on Age, Sex, and Performance. Front. Public Health.

[4] Fu Q., Levine B.D. (2013). Exercise and the autonomic nervous system. Handb. Clin. Neurol..

[5] Rice T., An P., Gagnon J., Leon A.S., Skinner J.S., Wilmore J.H., Bouchard C., Rao D.C. (2002). Heritability of HR and BP response to exercise training in the HERITAGE Family Study. Med. Sci. Sports Exerc..

[6] van de Vegte Y.J., Tegegne B.S., Verweij N., Snieder H., der Harst D.V. (2019). Genetics and the heart rate response to exercise. Cell Mol. Life Sci..

[7] Fisher J.P. (2013). Autonomic control of the heart during exercise in humans: Role of skeletal muscle afferents. Exp. Physiol..

[8] Brawner C.A., Keteyian S.J., Ehrman J.K. (2002). The relationship of heart rate reserve to VO_2_ reserve in patients with heart disease. Med. Sci. Sports Exerc..

[9] Mendez-Villanueva A., Landaluce J.P., García B.F., Terrados N., Bishop D. (2010). Inaccuracy of the HR reserve vs. V˙O2 reserve relationship during prone arm-paddling exercise in surfboard riders. J. Physiol. Anthropol..

[10] Chwalbínska-Moneta J., Krysztofiak F., Ziemba A., Nazar K., Kaciuba-Uściłko H. (1996). Threshold increases in plasma growth hormone in relation to plasma catecholamine and blood lactate concentrations during progressive exercise in endurance-trained athletes. Eur. J. Appl. Physiol. Occup. Physiol..

[11] Fisher J.P., Young C.N., Fadel P.J. (2015). Autonomic Adjustments to Exercise in Humans. Compr. Physiol..

[12] Wasserman K., Whipp B.J. (1975). Excercise physiology in health and disease. Am. Rev. Respir. Dis..

[13] Thomas J.R., Nelson J.K., Silverman S.J. (2015). Research Methods in Physical Activity.

[14] Garber C.E., Blissmer B., Deschenes M.R., Franklin B.A., Lamonte M.J., Lee I.-M., Nieman D.C., Swain D.P. (2011). Quantity and Quality of Exercise for Developing and Maintaining Cardiorespiratory, Musculoskeletal, and Neuromotor Fitness in Apparently Healthy Adults: Guidance for Prescribing Exercise. Med. Sci. Sports Exerc..

[15] Policarpo Barbosa F., Cruz M.S. (2016). Protocolo de rampa vs. escalonado: Análise do consumo de oxigênio e frequência cardíaca em jovens. Revista Brasileira de Ciência e Movimento.

[16] Policarpo Barbosa F., Silva P.E., Guimarães A.C., Pernambuco C.S., Dantas E.H. (2020). Prediction of maximum oxygen uptake through incremental exercise testing using ventilometry: A cross-sectional study. Braz. J. Phys. Ther..

[17] Grigaliūnienė A., Ramonas A., Celutkienė J., Sileikienė V., Rudys A., Juocevičius A., Laucevičius A. (2013). Cardiorespiratory parameters of exercise capacity in a healthy Lithuanian population: The pilot study. Hell. J. Cardiol. HJC Hell. Kardiol. Ep..

[18] Panton L.B., Graves J.E., Pollock M.L., Garzarella L., Carroll J.F., Leggett S.H., Lowenthal D.T., Guillen G.J. (1996). Relative Heart Rate, Heart Rate Reserve, and VO_2_ During Submaximal Exercise in the Elderly. J. Gerontol. Ser. A Boil. Sci. Med. Sci..

[19] Karvonen M.J., Kentala E., Mustala O. (1957). The effects of training on heart rate; a longitudinal study. Ann. Med. Exp. et Boil. Fenn..

[20] Kruel L.F.M., Beilke D.D., Kanitz A.C., Alberton C.L., Antunes A.H., Pantoja P.D., Da Silva E.M., Pinto S.S. (2013). Cardiorespiratory Responses to Stationary Running in Water and on Land. J. Sports Sci. Med..

[21] Brum P.C., Forjaz C.L.D.M., Tinucci T., Negrao C.E. (2004). Adaptações agudas e crônicas do exercício físico no sistema cardiovascular. Rev. Paul Educ. Fís..

[22] Astrand P., Rodahl K., Dahl H.A., Stromme S.B. (2003). Textbook of Work Physiology: Physiological Bases of Exercise.

[23] Pendergast D., Cerretelli P., Rennie D.W. (1979). Aerobic and glycolytic metabolism in arm exercise. J. Appl. Physiol..

[24] Silva L.R.B., Gentil P.R.V., Beltrame T.B., Filho M.A.B., Alves F.M., Silva S.M., Pedrino G.R., Ramirez-Campillo R., Coswig V., Rebelo A.C.S. (2019). Exponential model for analysis of heart rate responses and autonomic cardiac modulation during different intensities of physical exercise. Soc. Open Sci..

[25] Iannetta D., Keir D.A., Fontana F.Y., Inglis E.C., Mattu A.T., Paterson D.H., Pogliaghi S., Murias J.M. (2021). Evaluating the Accuracy of Using Fixed Ranges of METs to Categorize Exertional Intensity in a Het-erogeneous Group of Healthy Individuals: Implications for Cardiorespiratory Fitness and Health Outcomes. Sports Med..

[26] Ferri Marini C., Sisti D., Leon A.S., Skinner J.S., Sarzynski M.A., Bouchard C., Rocchi M.B.L., Piccoli G., Stocchi V., Federici A. (2021). HRR and V˙O2R Fractions Are Not Equivalent: Is It Time to Rethink Aerobic Exercise Prescription Methods?. Med. Sci. Sports Exerc..

